# Multiple system atrophy with oculomotor abnormalities as a prominent manifestation: A case series

**DOI:** 10.1097/MD.0000000000034008

**Published:** 2023-06-23

**Authors:** Yuanxiao Wei, Ju Chen, Cancan Lu, Yijing Jiang, Zhiqiang Liu, Wenzhao Zhang, Liqun Fang

**Affiliations:** a The Fourth Affiliated Hospital of Harbin Medical University, Harbin, China; b The Fourth Affiliated Hospital of Harbin Medical University, Harbin, China; c The Fourth Affiliated Hospital of Harbin Medical University, Harbin, China; d The Fourth Affiliated Hospital of Harbin Medical University, Harbin, China; e The Fourth Affiliated Hospital of Harbin Medical University, Harbin, China; f The Fourth Affiliated Hospital of Harbin Medical University, Harbin, China; g The Fourth Affiliated Hospital of Harbin Medical University, Harbin, China.

**Keywords:** multiple system atrophy, oculomotor abnormality, vertigo

## Abstract

Multiple system atrophy (MSA) is a group of adult-onset sporadic neurodegenerative diseases, mainly classified as MSA-C and MSA-P types. Due to the diversity of clinical symptoms, diagnosis faces a significant challenge. In the present case, we report a patient with isolated vertigo as the first presentation and abnormalities of the oculomotor system as the characteristic manifestations.

**Case concern:**

A 64-year-old male had dizziness for 1 year, aggravated for 4 months, with accompanying symptoms of unsteady walking. Physical examination revealed spontaneous nystagmus, abnormal ataxic movements, and a broad basal gait. Video nystagmography revealed saccade intrusions and macrosaccadic oscillations, and opsoclonus. Magnetic resonance imaging (MRI) was unremarkable early, and positron emission tomography-computed tomography (PET-CT) announced a reduction in the volume of the cerebellum and brainstem.

**Diagnosis:**

The diagnosis of the possibility of MSA type-C, peripheral neuropathy, hypertension, and lacunar cerebral infarction was performed.

**Conclusion:**

Atypical early clinical presentation may lead to delays, and identifying the critical problem through the patient simple clinical status requires long-term clinical experience and various ancillary examination tools.

## 1. Introduction

Multiple system atrophy (MSA) is a sporadic neurodegenerative disease with unknown etiology. It is an oligodendrocyte α-synucleinopathy characterized by autonomic dysfunction, Parkinson symptoms, cerebellar ataxia, and pyramidal tract symptoms.^[1]^

Vertigo-related diseases are common and frequently occur in the outpatient and emergency neurology department. Their complex etiology involves infection, immune-mediated, vascular, tumor, demyelination, and other factors. Clinical diagnosis is complicated when the only physical symptom is isolated vertigo, and the physical sign is oculomotor abnormalities. This paper reviewed the entire diagnosis and treatment process for patients with MSA, with oculomotor abnormalities being the notable performance.

## 2. Case report

A 64-year-old male has been experiencing walking vertigo for a year, resolving independently. In the past 4 months, the patient felt slightly unstable walking which worsened, occasionally with inflexible finger movements, without limb weakness. He had been treated in several hospitals and received neuropsychological treatment, such as antianxiety drugs, but his symptoms did not improve. The patient had a previous history of hypertension for more than 1 year, a record of smoking for more than 30 years, averaging 20 cigarettes per day, and social drinking for more than 30 years, about half a pound each time. He denied a history of motion sickness and any genetic disease in the family.

Physical examination of the nervous and vestibular systems revealed that he was conscious, speaking fluently and smoothly, his eye movements were regular, and his regulation and vergence reflexes were normal, but his spontaneous nystagmus was positive. Biceps, triceps, knee tendon reflexes, and Achilles tendon reflexes diminished bilaterally; bilateral Babinski signs were negative. He walked with a broad basal gait and poor straight-line walking; the Romberg test was unstable standing with eyes open and closed, closed eyes more obviously; the Fukuda test skewed to the right. Blood pressure was in the normal range in the prone position.

Cerebrospinal fluid examination showed protein qualitative 2+, cell count 0.028 × 10^9^ (0-0.02 × 10^9^), protein 2.05 g/L (0.2–0.4), chlorine 118.3 mmol/L (120–130), IgA 0.112 g/L (0–0.002).

Neurological paraneoplastic antibodies in serum and cerebrospinal fluid showed anti-GAD65 antibody IgG, anti-Zic4 antibody IgG, anti-SOX1 antibody IgG, anti-Ma2 antibody IgG, anti-Ma1 antibody IgG, anti-Amphiphysin antibody IgG, anti-CV2 antibody IgG, anti-Ri antibody IgG, anti-Yo antibody IgG, anti-Hu antibody IgG were all negative.

**Figure 1.**
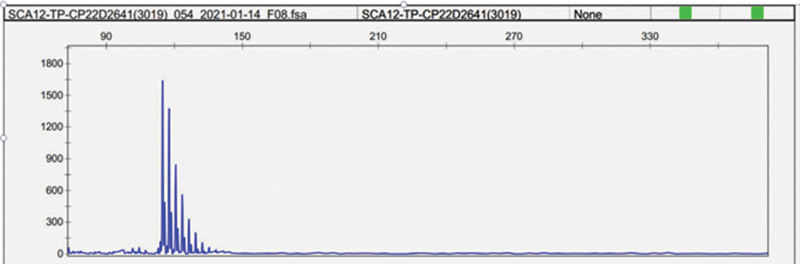
Gene fragment analysis.

Spinocerebellar ataxia (SCA) 12 gene fragment analysis showed that the number of CAG repeats of SCA 12-related genes was 16, which belonged to the normal range, and no abnormal alleles were found (Fig. 1).

Video nystagmography showed the oculomotor system was abnormal with spontaneous nystagmus, saccade intrusions, interval macrosaccadic oscillations, and opsoclonus (Supplementary video 1, http://links.lww.com/MD/J121). Visual tracking eye movement response: type II. The left side of canal paresis: decreased by 13% (0%–10%) (Review results after 7 days as before).

Nerve conduction velocity examination found peripheral nerve damage in both lower extremities.

Residual urine test showed bladder residual urine volume was about 30ml.

The upright tilt test was negative.

**Figure 2.**
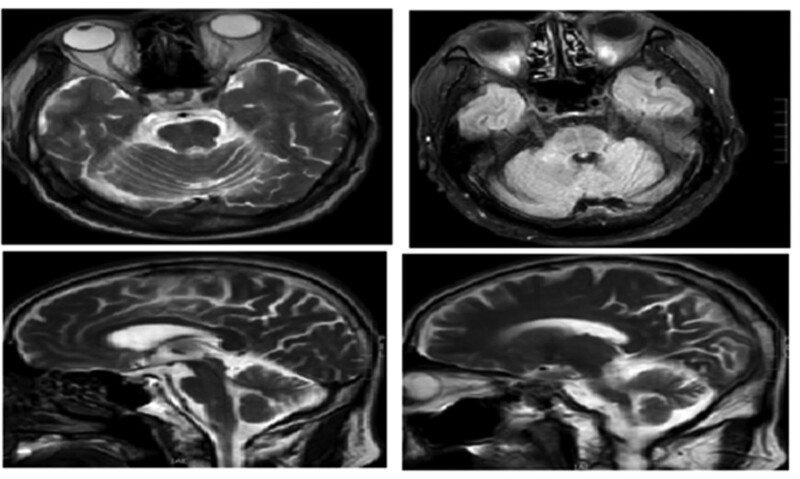
The cranial MRI did not show cerebellar and Brainstem atrophy. MRI = magnetic resonance imaging.

**Figure 3.**
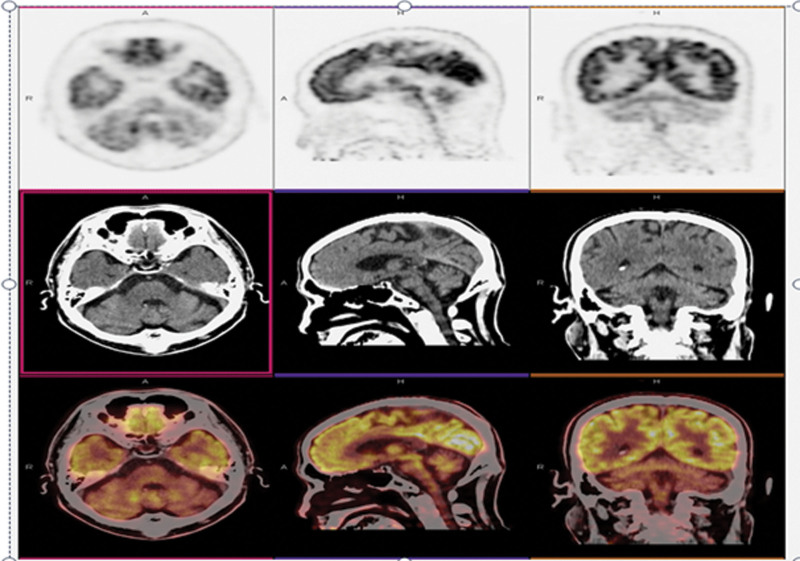
PET-CT showed diffuse slightly reduced metabolism in bilateral cerebellar hemispheres, the reduced volume of the Brainstem and cerebellum. PET-CT = positron emission tomography-computed tomography.

The cranial 3.0T magnetic resonance imaging (MRI) (Fig. 2) revealed a few lacunes without brainstem, cerebellar, or cerebral atrophy; the cervical spine MRI revealed no obvious abnormality. positron emission tomography-computed tomography (PET-CT) (Fig. 3) revealed that the metabolism of the bilateral cerebellar hemispheres was diffusely reduced, especially on the left side, with a metabolic reduction of approximately 31% compared to the contralateral side; the brainstem and cerebellum reduced in volume, the anterior border of the pons was smooth, the fourth ventricle dilated, the pontine cistern and the bilateral pontocerebellar angle cistern enlarged; the supratentorial ventricles mildly enlarged, and the cerebral sulcus and cerebral fissure widened in some parts of the brain.

## 3. Discussion

In this case, the primary symptom is chronic isolated vertigo; other symptoms include abnormalities of the oculomotor system, such as saccade intrusions, interval emergence of macrosaccadic oscillations, and opsoclonus; there is also a modest degree of cerebellar ataxia. In contrast, the patient cranial 3.0T MRI came out negative, suggesting that oculomotor abnormalities may be more sensitive than MRI tests for some neurodegenerative diseases.

While clinical saccade intrusions are often mistaken for patient noncooperation, constant excessive saccade intrusions or oscillations are mostly an abnormal state. Excessive saccade intrusions result in decreased fixation and are a manifestation of fixation disorder. The reflex saccade initiation center of the brainstem has 3 neurons, including impulse neurons that send impulses to initiate saccade, tonic neurons that send impulses to maintain the eye in the new position it arrives at, and terminating neurons that send inhibitory impulses to pulse neurons to terminate the impulse neurons. A lesion in the terminating neuron or a lesion in the inhibitory pathway mediated by the cortical oculomotor center via the basal ganglia will not inhibit the impulse neurons or excitatory neurons, both of which can cause saccade oscillation.^[2]^ The cerebellum cannot effectively inhibit or optimize the brainstem oculomotor function, resulting in the instability of the brainstem oculomotor center, which is also the cause of saccade invasions or oscillations.

Localization analysis: The patient showed intermittent macrosaccadic oscillations and opsoclonus, so the brainstem (burst neuron deactivation) and cerebellum (fastigial nucleus deactivation) were localize^[3]^; since the Romberg sign was positive for open and closed eyes and could not complete walking in a straight line, the cerebellar cerebellum considered. Additionally, PET-CT found the brainstem and cerebellum to reduce in volume.

Qualitative analysis: diagnosis of probable multisystem atrophy - type C. Supporting points include an elderly male with insidious onset and slow progression, manifested by vertigo, unsteady walking, intermittent abnormalities of the oculomotor system, and ataxic manifestations, suspicious cross signs in the brainstem on MRI. PET-CT revealed it reduced brainstem and cerebellar volume and bilateral cerebellar hemisphere hypometabolism, with the left side being the most predominant. Unsupportive points included the absence of significant autonomic symptoms, such as urinary frequency, urinary urgency, reduced sexual function, normal range of blood pressure in the prone position, negative upright tilt test, and normal sympathetic skin response. Although the residual bladder volume is about 30 ml, studies have shown that a residual urine volume >100 ml contributes to the diagnosis of MSA.

The following 2 conditions are primarily part of the differential diagnosis. Firstly, SCA is a heterogeneous collection of autosomal dominant neurodegenerative diseases, with cerebellar ataxia and neurodegenerative alterations in the cerebellum and efferent pathways as a joint presentation. However, the patient experienced cerebellar ataxia symptoms, and the diagnosis of the condition not now support by the patient lack of family history, the absence of spinal cord lesions, and his negative spinal cerebellar ataxia gene fragment analysis results. Secondly, the paraneoplastic neurological syndrome is suspected because the patient had cerebellar ataxia, a high cell count, and protein count in the cerebrospinal fluid. However, the disease was not considered at this time because the patient tumor-related test results were thus far negative. The effects of eleven neurological paraneoplastic antibodies in serum and cerebrospinal fluid were likewise adverse.

## 4. Conclusion

This case presented clinically with only isolated vertigo symptoms, with signs of abnormal eye movements and mild cerebellar ataxia, which very quickly led to misdiagnosis in the absence of abnormalities on conventional imaging. Many nystagmus and nystagmus-like movements manifest specific underlying pathological mechanisms, reflecting the correlation between phenomenon and essence. Therefore, we need to look at the spirit through the clinical phenomenon, learn to dissect and identify the etiology of vertigo layer by layer and verify it with the help of various methods such as imaging, genetics, and immunohistochemistry, which can reveal where the true etiology of the disease lies.

## Author contributions

**Conceptualization:** Ju Chen.

**Formal analysis:** Cancan Lu.

**Investigation:** Yijing Jiang.

**Validation:** Zhiqiang Liu.

**Visualization:** Wenzhao Zhang.

**Writing – original draft:** Yuanxiao Wei.

**Writing – review & editing:** Liqun Fang.

## Supplementary Material


